# Profiles of teacher–child interaction quality in groups of 3-year-old children in Quebec and France

**DOI:** 10.1007/s43545-021-00266-8

**Published:** 2021-10-22

**Authors:** Maude Roy-Vallières, Nathalie Bigras, Annie Charron, Caroline Bouchard, Andréanne Gagné, Philippe Dessus

**Affiliations:** 1 Équipe de recherche Qualité des Contextes Éducatifs de la Petite Enfance, Faculté des Sciences de l’Éducation, Université du Québec à Montréal, Montréal, QC Canada; 2 Faculté des Sciences de l’Éducation, Université Laval, Québec, QC Canada; 3 Laboratoire de Recherche sur les Apprentissages en Contexte (LaRAC), Université Grenoble Alpes, Grenoble, France

**Keywords:** Early childhood, Adult–child interaction quality, Structural quality, Latent profile analysis, Classroom Assessment Scoring System

## Abstract

Theory and studies support that educational quality may differ according to socio-political context even in states with similar cultures. Based on a secondary analysis of data, this study aims at identifying latent profiles of adult–child interaction quality in groups of three-year-old children in Quebec’s (Canada) early childhood centers and France’s kindergarten classrooms using the CLASS Pre-K. This study also aims to explore existing associations between identified profiles, socio-political contexts, and structural characteristics (staff qualifications, ages, group size). Latent profile analyses showed four interaction quality profiles, namely a high-quality profile (HQ), a medium–high-quality profile (MHQ), a medium quality profile (MQ), and a medium–low-quality profile (MLQ). The scores of the three CLASS Pre-K domains associated with identified profiles show a higher average interaction quality in Quebec compared with France, suggesting a more favorable sociocultural context for interaction quality in Quebec. As for characteristics of structural quality, analyses suggest that the group size variable is significantly associated with scores of interaction quality, with the HQ and the MHQ profiles showing a significantly lower group size than the MQ and MLQ profiles. Age is also significantly associated with profiles, exhibiting a general trend of younger participants found in higher quality profiles. Courses of action to enhance French policies are discussed.

## Introduction

In the education field, it is widely accepted that early childhood is of critical importance in children’s development and educational success (April et al. 2018; Bouchard et al. 2017; Johnson 2021; Yoshikawa et al. 2018). The brain’s accelerated growth in that period of time helps children learn a variety of things at an impressive speed (Bouchard 2019; Jirout et al. 2019). Children’s ability to learn are affected not only by their genetic background, but also by experiences (Simard et al. 2013; Taylor and Boyer 2020; Yoshikawa et al. 2013). Living new and diverse experiences will allow children’s neurological development to speed up which, in turn, enhances motor, socioemotional, language, and cognitive development (Hassinger-Das et al. 2017). Consequently, high-quality educational environments that offer experiences adapted to children’s ability and interests have several advantages over lower quality contexts: prevention of school failures, reduction of behavioral problems, and improved overall development (April et al. 2018; Garcia et al. 2021; van Huizen and Platenga 2018). Thus, attending high-quality educational childcare services (ECS) in early childhood seems to be a protective factor for children who have few stimulation opportunities at home, as evidenced by studies linking attendance of quality ECS with higher developmental gains in children living in low-socioeconomic backgrounds (e.g., Burchinal et al. 2010; Laurin et al. 2019; van Huizen and Plantenga 2018). At the same time, low-quality ECS could exacerbate some developmental delays and learning difficulties. Thereby, there is a need for high-quality educational contexts to support the learning of children from all socioeconomic backgrounds (Organisation for Economic Co-operation and Development [OECD] 2015). Hence, several states, including France and Quebec, have set up quality assessment practices following this objective (OECD 2015).

### Educational quality in early childcare

Representations on the concept of quality and what accounts to a high-quality ECS have been known to depend on sociocultural values and contexts (Madani 2019; OECD 2015; Tobin 2005; Wolf 2018). However, many early childhood researchers conceptualize educational quality as having two main dimensions, namely structural quality and process quality (Bigras et al. 2020; Slot 2018).

On the one hand, structural quality involves factors in the educational context that affect adult work. It includes, in particular, adult–child ratio, group size, and staff training and experience (Bigras et al. 2020; Ruopp 1979). Although studies have shown that additional training, or professional development, is frequently and strongly associated with child development measures (Egert 2015; Jensen et al. 2017; Markussen-Brown et al. 2017), results for other components of structural quality are mixed (for a meta-analysis, see Slot 2018). Some authors have thus put forward the hypothesis that structural quality might indirectly impact children’s development by moderating the effects of process quality (Melhuish et al. 2015). Although few studies have investigated this possibility (e.g., Markussen-Brown et al. 2017; Slot et al. 2018), their results seem to support this hypothesis. However, more studies are needed to confirm this relationship (Slot 2018), which could guide research on ways to improve interaction quality through policies and regulations.

On the other hand, process quality relates to factors that influence children’s experience in ECS, specifically classroom organization, adaptation of activities to meet children’s needs, and adult–child interaction quality. Recent studies in early childhood education have been mostly concerned with the effects of adult–child interaction quality on child development (Melhuish et al. 2015). Indeed, Sabol et al. (2013), in a meta-analysis of two comprehensive United States studies, have shown that this process quality variable, as measured by the *Classroom Assessment Scoring System* (CLASS; Pianta et al. 2008), has the best predictive value for children’s cognitive, language, and socioemotional development. Accordingly, interaction quality is a key variable to measure to ensure a positive effect of ECS on child development (Choi et al. 2019; Mortensen and Barnett 2015; Ulferts et al. 2019; Williford et al. 2013).

The prominent role of adult interactions on child development can be explained by Hamre and Pianta’s (2007) *Teaching Through Interactions* model, which states that the sense of security originating from a sensitive and stable relationship with an adult allows children to manage their emotions, take risks, and acquire abilities and skills. Several empirical studies have offered support for this model by showing gains in social and academic skills linked to adult–child interaction quality (e.g., Choi et al. 2019; Conroy et al. 2015; Hu et al. 2019; Leyva et al. 2015).

While educational quality can be explained in plain terms of structural and process quality, its operationalisation is not always straightforward. In fact, people will view quality differently according to what they value for education and children’s well-being (Bouve 2017). For example, while Quebec and France show similarities which could lead one to expect comparable levels of educational quality, such as being considered WEIRD (Western, Educated, Industrialized, Rich, and Democratic) cultures (Henrich et al. 2010) and belonging to the same French-speaking community, studies have shown that they favor different educational approaches (Anders 2015; Bigras et al. 2020; Tazouti et al. 2011). In turn, both approaches imply distinct educational methods and program content, possibly leading to discrepancies in how educational quality is applied in their ECS.

A meta-analysis from Vermeer et al. (2016) also supports this claim, showing that interaction quality scores would vary widely around the world, even though most of the selected countries could fall under the WEIRD culture category. The authors speculated that discrepancies could be explained by socio-political characteristics, namely, government policies regulating physical environments, subsidizing or quality rating, and improvement systems, among others. Hence, there are indicators that differences in educational approaches leading from variations in socio-political contexts might affect children’s daily experiences in ECS, which we aimed to explore in the similar WEIRD cultural contexts of France and Quebec.

### Educational quality in France and Quebec

#### France’s approach to educational quality

In France, kindergarten schools, whose attendance has been compulsory since 2019, are open to children between the ages of 3 and 5, as well as from 2 years in vulnerable contexts (Ministère de l’Éducation Nationale et de la Jeunesse 2020a). These establishments take a school readiness approach, emphasizing adult-led activities (Siraj-Blatchford et al. 2002; Stipek 1991; Weikart 2000) and involving school content, such as learning letters, numbers, and writing (Brougère 2002). Particularities of this approach can be seen in the schools’ structural and process quality.

Indeed, group size is usually of one adult for 25 children (Tobin 2005), while about a third of kindergarten teachers are supported by a specialized assistant (*Agent territorial spécialisé des écoles maternelles* in French, or ATSEM) who helps with specific tasks. It may be noted here the similarity with the French elementary schools, which have a mean of 22.7 pupils per classroom (Ministère de l’Éducation Nationale et de la Jeunesse 2020b).

As for initial training, French kindergarten teachers follow a five-year university course (bac plus 5) and must pass the recruitment competition for school teachers (Valette 2019), which is a two-phase exam comprising several written and oral tests, allowing them to work in both kindergarten and elementary schools. Consequently, they are not specifically trained for early childhood education (Cochran 2011; Rayna 2004). In recent years, teachers pursuing a teaching degree, namely the *Certificat d’aptitude aux fonctions d’instituteur ou de professeur des écoles maître formateur* (CAFIPEMF), have had the opportunity to choose a specialty in kindergarten teaching as a way to complete their training (Ministère de l’Éducation nationale et de la jeunesse 2015). Despite this, the reports of the CAFIPEMF juries point out that only 10% of trainee teachers admitted to the certificate choose this option (e.g., Académie de Nantes 2019). For their part, ATSEMs have a level 4 diploma for the completion of the French high school, equivalent to a collegial diploma in Quebec.

With regard to process quality, the educational program in France emphasizes the development of communication skills through expressing, describing, and writing initiation activities (OECD 2004). Nevertheless, the child is expected to develop these skills independently through play and sensory experiences (Cochran 2011; Vitali 2007). Thus far, a number of studies have addressed the effects of French kindergarten school attendance on children’s development and academic achievement, with mixed results (Ben Ali 2012; Caille 2001; Florin 2007; Goux and Maurin 2010; West 2016). However, to the authors’ best knowledge, studies have yet to measure interaction quality in kindergarten schools, which is the variable most strongly associated with children’s development and overall educational success (Sabol et al. 2013). In that respect, variables of structural and process quality in French kindergarten schools remain to be explored, limiting our comprehension of what influences children’s development in France’s school system.

#### Quebec’s approach to educational quality

In Quebec, educational services targeting children from 3 to 5 years old concern primarily regulated child care, that is, services acknowledged by the *Ministère de la Famille* and in accordance with the Educational Childcare Act, including early childhood centers (ECC, officially named CPEs for *Centre de la petite enfance*) which accommodate a third of Quebec children between the ages of 12 months and 4 years (Ministère de la Famille 2017). In ECC, holistic development of the child is promoted. This approach excludes assessment of children’s academic skills (OECD 2006) and influences structural and process quality.

Thus, among variables contributing to the ECC structural quality, there is an adult–child ratio of 1:8 in groups of three-year-old children. This low ratio provides opportunities for individualized interactions between adults and children (Dalgaard et al. 2020).

In terms of training, approximately 80% of educators working in ECC hold a college diploma (DEC, for *Diplôme d’études collégiales*) or a college studies certificate (AEC, for *Attestation d’études collégiales*) (Rousseau et al. 2016). Skills and content covered in their training courses focus on educational practices that consider children’s needs and interests (Gouvernement du Québec 2000). In the case of the AEC, training includes fewer course credits (1800 h) and requires three years of proven experience in an ECS acknowledged by the *Ministère de la Famille* for graduation. It must be noted that the DEC and AEC in early childhood education are equivalent to the French baccalaureate and allow access to university education (Ministère de l’Europe et des Affaires étrangères 2017).

With regard to process quality, ECC’s educational program (Ministère de la Famille 2019) recommends techniques to support children’s holistic development, such as promoting play-based learning. Educators are encouraged to support children’s learning using scaffolding and positive interactions (Conseil supérieur de l’éducation [CSE] 2012). In this manner, studies carried out in Quebec have reported medium–high levels of process quality in ECC (Bigras et al. 2010; Bouchard et al. 2021; Drouin et al. 2004; Institut de la statistique du Québec 2015; Japel et al. 2005). These studies also indicate that ECC are the ones offering the highest levels of process quality in comparison to other Quebec ECS, such as private and family daycare. Nevertheless, with one exception (Bouchard et al. 2021), these studies have not measured interaction quality using the CLASS, restricting both the predictive power of results in respect to child development (Sabol et al. 2013) and their comparison with other international contexts (e.g., Cloney et al. 2017; Hu et al. 2016; Leyva et al. 2015; Stuck et al. 2016).

Considering the above elements, a double research problem exists regarding educational quality of ECS in France and Quebec. First, the results of structural and interaction quality studies in these ECS seem incomplete on both sides. Especially for French kindergartens, data are scarce on the variables of structural and interaction quality that can support children’s development. In addition, to the best of the authors’ knowledge, there has been no study comparing the Quebec and French educational contexts in terms of structural and process quality that could provide information on which educational approach makes it easier to achieve high-quality ECS in WEIRD cultured states. Such a comparison could also make it possible to identify characteristics and educational practices specific to Quebec’s or France’s socio-political contexts that are particularly relevant in supporting children’s development or needing improvement. This would ultimately help improve overall childcare quality by building on the states’ respective strengths to provide lasting solutions to the identified weaknesses.

### Comparison of educational quality in Quebec and French ECS

To bridge this gap, a comparative study of structural and interaction quality (process quality) in three-year-old groups of children in French kindergartens and Quebec ECC was conducted in 2017–2018 (Bigras et al. 2020). This study used the CLASS Pre-K measure (Pianta et al. 2008) to observe interaction quality in three main areas, namely emotional support, classroom organization, and instructional support. Results suggested that interaction quality was significantly higher in Quebec’s ECC than in France’s kindergartens. Furthermore, ECC generally had higher mean scores in the CLASS Pre-K domains than the average scores reported by the creators of the CLASS Pre-K measure (Pianta et al. 2008), whereas for kindergartens it was lower overall.

However, although the mean is the central trend value which generally represents most faithfully the distribution of data (Haccoun and Cousineau 2010), it may be limited in certain cases. For example, the mean’s value is not always found in the sample’s data. In addition, means can create inadequate representations of reality, especially when the distribution of data is not homogeneous (Haccoun and Cousineau 2010).

### Clustering analysis techniques

As proposed by Salminen et al. (2012), a variable-centered analysis, as reported by Bigras et al. (2020), does not reveal specific trends in a population gathering people with similar profiles. To reveal those trends, one must rather use a person-centered approach, which will allow each participant to be associated with a group displaying specific characteristics. In early childhood research, a number of studies have identified educational quality profiles from CLASS Pre-K scores using two different methods, that is, cluster analysis and latent profile analysis (Bouchard et al. 2021; Hoang et al. 2019; Hu et al. 2016; LoCasale-Crouch et al. 2007; Salminen et al. 2012). These analyses allow researchers to refine results by providing identified profiles’ prevalence in the sample and by associating these profiles with other criteria, such as socio-political context (e.g., France’s and Quebec’s). A review of studies in early childhood using person-centered analyses is presented in the next paragraphs, while Table 1 synthetizes this information.

**Table 1:** Synthesis of research in early childhood using person-centered analyses

Author	Technique	Sample	Profiles identified	Dominant profile	Discriminating factors
LoCasale-Crouch et al. (2007)	Cluster analysis	692 groups	5	Median quality	Variations in overall scores
Bouchard et al. (2021)	Cluster analysis	15 groups	3	Lowest quality	Instructional support scores
Salminen et al. (2012)	Latent profile analysis	49 groups	4	Highest quality	Emotional support scores
Hu et al. (2016)	Latent profile analysis	180 groups	4	Median quality	Variations in overall scores
Hoang et al. (2019)	Latent profile analysis	57 groups	3	Highest quality	Variations in overall scores

In 2007, using a cluster analysis, LoCasale-Crouch et al. identified five profiles of interaction quality among 692 teachers in the United States. These profiles were mainly discriminated by variations in scores between a high and a low level (LoCasale-Crouch et al. 2007). Most teachers were grouped in the median profile, providing a high level of emotional support and a medium level of instructional support. The researchers also noted that participants in the higher profiles had more years of preschool experience as well as a reduced adult–child ratio, and those in the lower profiles showed more hours in class per day and pupils from disadvantaged socioeconomic backgrounds (LoCasale-Crouch et al. 2007).

Using the same technique, Bouchard et al. (2021) identified three profiles of interaction quality in 15 groups of 4-year-old children in Quebec ECC. Profiles were mainly differentiated in terms of instructional support scores, most of the groups finding themselves in the lowest profile. However, scores of emotional support and classroom organization were at least in the medium–high level for all profiles (Bouchard et al. 2021).

Concurrently, Salminen et al. (2012), Hu et al. (2016), and Hoang et al. (2019) performed latent profile analyses on CLASS Pre-K scores. In a study by Salminen et al. (2012), the researchers found four quality profiles in 49 preschool classrooms in Finland. The profiles were discriminated in large part by emotional support scores (Salminen et al. 2012). In addition, teachers in the lowest quality profile had less teaching experience and training in literacy teaching, even though 53% of their sample was associated with the highest quality profile.

Similarly, Hu et al. (2016) identified four profiles of interaction quality in 180 preschool classrooms in China. Most of their sample gathered in a median profile with high scores for emotional support and classroom organization, as well as a low score for instructional support. In addition, teachers with higher professional characteristics, such as more experience or ongoing training, were more likely to find themselves in high-quality profiles, while classrooms in low-socioeconomic areas were more likely to end up in low-quality profiles. No association was found between the profiles and participation in a specialized early childhood training program (Hu et al. 2016).

Finally, Hoang et al. (2019) identified three interaction quality profiles in 57 Vietnamese preschool classrooms, where the majority (75.4%) of teachers grouped in the highest quality profile. Hoang et al. (2019) also noted a significant association between teacher experience and quality scores, more experienced teachers being more likely to end up in the high-quality profile.

In summary, the number and prevalence of identified profiles vary according to each study. As the same analyses were generally used in the studies, this difference could be explained in part by socio-political context. Indeed, Salminen et al. (2012) suggested that variations in the homogeneity of teachers’ initial training could explain the differences in the number of profiles, while Hoang et al. (2019) have noted that discrepancies in children’s behavior relating to nationality may influence overall CLASS Pre-K scores.

In that regard, while both WEIRD French-speaking societies, the distinct socio-political contexts in France and Quebec lead us to think that the number and prevalence of profiles might be different between the two samples. Furthermore, studies seem to indicate that professional experience is associated with the interaction quality profiles, although results regarding specialization in early childhood are mixed (Hu et al. 2016). Adult–child ratio and socioeconomic background have also been linked to the identified profiles (Hu et al. 2016; LoCasale-Crouch et al. 2007). The authors will therefore identify if such trends exist in the sample from data collected on structural quality variables.

### Aims of the present study

Therefore, the present study aims at identifying latent adult–child interaction quality profiles among French teachers and Quebec educators and their associations with structural variables involving a secondary analysis of Bigras et al.’s (2020) data. These analyses will deepen the understanding of the quality levels observed in groups of 3-year-old children in France and Quebec, determine the prevalent adult–child interaction quality profiles in the combined sample, and identify their links with specific structural variables. In return, these results will make it possible to understand which structural variables should be modified by new regulations in order to improve overall quality in both contexts. Based on the presented previous studies using either cluster or latent profile analyses in the early childhood context, the authors expect to find between three and five interaction quality profiles. Moreover, considering Bigras et al.’s (2020) data, the authors hypothesize that participants from Quebec will likely be classified in higher quality profiles, while participants from France could find themselves in the lower quality profiles. Finally, due to the distinct educational approaches of the French and Quebec contexts, and policies and regulations varying accordingly, it is expected that the variable of socio-political context (France’s or Quebec’s) will explain most of the affiliation in particular profiles.

## Method

### Participants

The sample is composed of 40 ECC educators in Quebec and 41 kindergarten teachers in France (including 37 females), all working with groups of 3-year-old children. Educators come from ECC in Montreal (Quebec, Canada) and were recruited from February to March 2017, whereas kindergarten teachers come from the Grenoble region (France) and were recruited from October 2017 to January 2018. Participating ECS serve populations with a similar rate of low-socioeconomic families, namely, 29% in Montreal and 28% in Grenoble. Following a random draw from regional lists of ECC (334) and kindergarten schools (74), made available by official authorities (*Ministère de la Famille* in Quebec and *Rectorat de l’académie de Grenoble in France*), ECS were contacted by email and then by phone in order to explain the study and obtain participants’ consent.

### Measures

#### Interaction quality observation tool

The *Classroom Assessment Scoring System* (CLASS; Pianta et al. 2008) Pre-K is an adult–child interaction quality evaluation tool designed for the assessment of educational contexts serving children from 3 to 5 years old. It is composed of three domains: (1) *Emotional Support* (positive climate, negative climate, sensitivity, regard for student perspective), (2) *Classroom Organisation* (behavior management, productivity, instructional learning formats), and (3) *Instructional Support* (concept development, quality of feedback, language modeling). Assessment using CLASS Pre-K involves a minimum of four observation cycles of 30 min each (20-min observation, 10-min coding). Each dimension is rated on a seven-point Likert scale (1–2 = low, 3–5 = medium, 6–7 = high) for each observation cycle. Domain scores are then computed in two steps: (1) creation of dimension composite scores from the average of the scores assigned after each observation cycle and (2) averaging dimension composite scores from the same domain to create domain scores. The instrument offers a good fidelity level with internal consistency varying from good to excellent, depending on the dimensions (*α* = 0.79 to 0.91) (Pianta et al. 2008). Research assistants (*n* = 9) were from Quebec and had achieved CLASS Pre-K certification after completing a mandatory two-day training course and passing an online exam. During data collection, a sample size corresponding to 15% of all observations was used to carry out inter-rater reliability measures in each of the contexts (Montreal and Grenoble), as recommended by Bujang and Baharim (2017). Average percentage agreement rates of 98% (Grenoble) and 89% (Montreal) have been obtained, corresponding to a high level of inter-judge agreement (Cicchetti 1994).

#### Structural quality questionnaire

Two questionnaires from Drouin et al. (2004) were used to measure structural quality in the two educational contexts. The first one was the *Self-Administered Questionnaire for the Educator (QP-1)*, composed of 27 questions divided under four topics: feelings regarding work environment and job satisfaction, personal background, education and training and, finally, working conditions, work experience, and professional development. The second one was the *Telephone Questionnaire for the Manager of the Day Care Centre or the Childcare Centre Facility (QP-2)* and was used to collect data on adult–child ratio, number of children in the group/service, and age of children. The interested reader will find descriptive analyses of these data in Bigras et al. (2020).

### Procedure

Data collection was carried out in Quebec ECC from February to May 2017 and in French kindergartens in March 2018. It included the assessment of interaction quality using the CLASS Pre-K tool and filling out of a questionnaire by educators/teachers on structural quality characteristics (training, experience, ratio, etc.). The study obtained certificates of ethics approval from two university ethics committees, namely, from UQAM’s committee (CERPE) and from the University of Grenoble Alpes’ committee (CERGA). In addition, the study was subject to the approval of the data protection correspondent for the legal and litigation department of the Grenoble Rectorate (decree of September 26, 2017).

### Statistical analyses

From this literature review, it is noted that early childhood studies favor the use of latent class analysis instead of standard cluster analysis. Although previously difficult to achieve due to the need for high processing power, latent class analysis has the advantage of being a model-based approach (Magidson and Vermunt 2002b). Hence, the analysis assumes that the data stem from a statistical model and allow for a less arbitrary choice of cluster criterion. Latent class analysis also offers easier handling of variables pertaining to separate measurement scales (Magidson and Vermunt 2002b). Although authors have suggested that a minimal sample size of *N* = 500 should be used in latent class analysis to provide sufficient statistical power (Finch and Bronk 2011), there is in fact no consensus in the literature. Park and Yu (2018) raised that the required sample size should vary depending on several factors, including the number of indicators and the class structure, while Nylund-Gibson and Choi (2018) suggested that a sample size of *N* = 30 could be sufficient in a model with distinct classes. In addition, Formann’s minimal rule indicates that a sample of *N* = 2^*k*^ (where *k* = number of variables) should be enough to provide satisfactory statistical power (Formann 1984). Hence, we used a multi-group approach to obtain a sufficient sample size to conduct a latent class analysis, while also investigating for relations between identified profiles and socio-political context as a way to distinguish between our two samples.

In order to identify profiles of interaction quality associated with educators’ and kindergarten teachers’ scores on the CLASS Pre-K, a latent profile analysis was performed according to Formann (1984). Latent profile analysis implies a finite mixture model. It considers that there exists an underlying categorical variable based on which individuals from in a population will form mutually exclusive latent profiles (Witherspoon et al. 2019). While true profile membership is unknown, it is inferred in the analysis using two parameter sets, namely, membership probability (likely distribution of profiles in the original population) and item-response means and variances, in this case CLASS mean scores and variances. It should be noted that the latent profile analysis does not require normal distribution or homogeneity as with traditional modeling techniques, allowing for less statistical bias (Magidson and Vermont 2002a). The analyses were carried out in the *Mplus* software, version 7.31 (Muthén and Muthén 1998).

First, we had to determine the number of profiles that would make up the model. Several indicators are used to assess relative fit of the latent profile model by comparing the current model with *k* profiles to an alternative model with *k − *1 profiles. These indicators are the Bayesian Information Criteria (BIC; Schwartz 1978), adjusted BIC (ABIC), Bayes Factor (BF), correct model probability (cmP), Bootstrap Likelihood ratio Test (BLRT; McLachlan and Peel 2004), and Vuong Lo Mendel Rubin LRT (VLMR-LRT; Vuong 1989). While lower BIC and ABIC values indicate higher model fit, non-significant BLRT and VLMR-LRT values suggest better model fit for the *k* − 1 profiles model (Nylund et al. 2007). Finally, higher cmP values and BF values over 10 show higher probability of the model being accurate (Masyn, 2013). While none of these tests have been shown to work equally under all modeling conditions, the BLRT and BIC indicators are recognized as the most reliable for determining model fit (Nylund et al. 2007), and thus take on more importance when choosing which latent profile model to retain.

It should also be noted that while the goal of the latent profile algorithm is to find the solution which best represents the data (global maximum), that is a solution in which the data are most probable, sometimes it will converge on a local maximum solution, which is close to but not quite the most representative for the data. Uebersax (2009) uses a metaphor of going up a mountain: “By proceeding constantly uphill, always taking the steepest slope, you will reach the top of whatever peak you are already on. However, the highest peak may actually be across a valley; to reach it, you would need to first go downhill, and then uphill again. Finding a global maximum can be difficult for most estimation algorithms, because their strategy is to move "uphill" at all times.” Hence, during the analysis, several starting values are generated and tested to avoid local maxima using an automatic option in *Mplus* 7.31. Finally, participants are associated with each profile according to the two sets of parameters discussed above that is membership probability and item-response means and variances. It should be noted that only domain scores were used in the analysis, as the higher number of dimensions would lead to lack of statistical power with our relatively low sample.

## Results

### Latent profile analysis results

Table 2 showcases the indicator tests’ results used to assess fit of the interaction quality latent profile models.

**Table 2:** Fit indices for interaction quality latent profile modeling

Nb of profiles	AIC	BIC	ABIC	Entropy	VLMR-LRT	LMR-LRT	BLRT
1	− 326.166	678.698	659.776	NA	NA	NA	NA
2	550.762	574.706	543.17	0.898	< 0.001	< 0.001	< 0.001
3	523.33	556.852	512.701	0.881	**0.0105**	**0.0133**	< 0.001
4	513.657	**556.757**	499.991	0.864	0.3155	0.3364	< 0.001
5	506.125	558.803	489.423	0.884	0.1408	0.1511	** < 0.001**
6	502.067	564.323	482.328	NA	0.0445	0.0525	0.1429

Values in bold indicate the preferred model for a given fit index

Three profile models were suggested based of the fit indices: the VLMR-LRT and the LMR-LRT suggested a three-profile model, while the BIC supported a four-profile model and the BLRT recommended a five-profile model. As discussed above, the BLRT and the BIC are more reliable indicators, thus the three-profile model was not retained. Considering the discrepancies between the BLRT and the BIC and lack of other significant fit indicators, the four-profile model was ultimately chosen for its more easily interpretable results.

### Model specifications

Regarding our multi-group (France and Quebec) sample’s interaction quality scores, the latent profile analysis revealed four profiles: a high-quality (HQ) profile, a medium–high-quality (MHQ) profile, a medium quality (MQ) profile, and a medium–low-quality profile (MLQ). Descriptive statistics of the three CLASS Pre-K domains’ scores associated with the generated profiles are presented in Table 3.

**Table 3:** Interaction quality profiles

Domains	HQ (*n* = 19)		MHQ (*n* = 17)		MQ (*n* = 27)		MLQ (*n* = 18)		Total (*n* = 81)	
	M	SD	M	SD	M	SD	M	SD	M	SD
Emotional Support	6.43	0.33	5.33	0.44	4.24	0.55	3.72	0.51	4.87	1.04
Classroom Organization	6.22	0.37	5.51	0.35	4.56	0.35	3.40	0.41	4.16	1.30
Instructional Support	3.11	0.62	2.69	0.48	2.39	0.38	1.72	0.32	2.20	0.58

#### Profile 1: High quality (HQ) (prevalence = 23.5%)

Participants in this profile (*n* = 19) stand out with high average scores for emotional support (6.43) and classroom organization (6.22), while the average instructional support score is in the medium–low range (3.11). It should be noted that the average scores of participants in profile 1 are higher than the average scores of other profiles. The average scores for the three domains of the CLASS Pre-K in this profile are also higher than the average scores reported by the creators of the tool (Pianta et al. 2008).

#### Profile 2: Medium–high-quality (MHQ) (prevalence = 21%)

This profile incorporates participants (*n* = 17) for whom emotional support and classroom organization display high average scores (5.33 and 5.51), but low average scores for instructional support (2.69). The average scores of participants associated with this profile are still higher than the total average scores for the sample and the CLASS Pre-K (Pianta et al. 2008).

#### Profile 3: Medium quality (MQ) (prevalence = 33.3%)

This profile covers a third of the sample’s participants (*n* = 27) and is characterized by a medium level average score for emotional support (4.24) and classroom organization (4.56), while displaying a low-level instructional support (2.39). Their scores are similar to the average scores reported by Pianta et al. (2008) except for a slightly higher instructional support.

#### Profile 4: Medium–low-quality (MLQ) (prevalence = 22.2%)

**Fig. 1:**
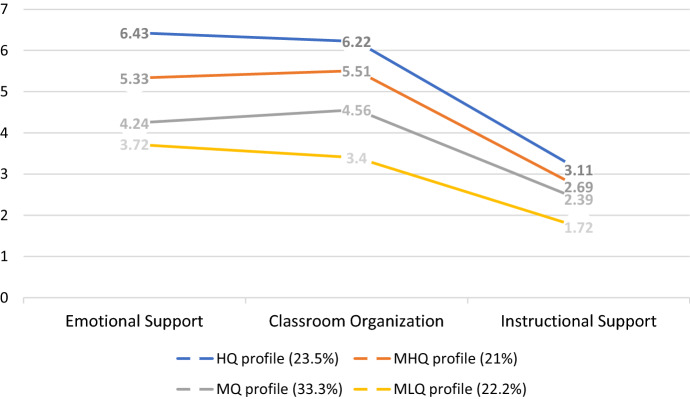
Interaction quality profiles

This last profile has the lowest average scores of the four profiles generated. It is characterized by medium–low emotional support and classroom organization scores (3.72 and 3.40) and a low instructional support score (1.72). The average scores associated with this profile are lower than the average scores reported by the creators of the CLASS Pre-K (Pianta et al. 2008). The profiles are visually represented in Fig. 1.

### Socio-political context

As for socio-political context, results of the descriptive and statistical analyses are presented in Table 4. A Chi-squared test was performed to verify if there was a significant affiliation to profiles depending on participants’ socio-political context that is whether they worked in Quebec or in France. A variation in subscript letters identifies a significant difference between countries.

**Table 4:** Profile affiliation likeliness by socio-political context

Profile	Quebec		France		Total	
	*n*	%	*n*	%	*n*	%
HQ	19_a_	47.5	0_b_	0	19	23.5
MHQ	15_a_	37.5	2_b_	4.9	17	21
MQ	4_a_	10	23_b_	56.1	27	33.3
MLQ	2_a_	5	16_b_	39	18	22.2
Total	40	100	41	100	81	100

Results in Table 4 show that affiliation to a profile is always significantly different between the two contexts. In fact, educators in Quebec are found significantly more in high-quality profiles (HQ and MHQ), while teachers in France are found in lower quality profiles (MQ and MLQ). It may be noted that only two Quebec educators find themselves in the lowest quality profile (MLQ), while no French teachers were classified in the HQ profile. Overall, this suggests that interaction quality is easier to attain in Quebec’s socio-political context than it is in France’s.

### Structural characteristics

Pertaining to the association of structural quality characteristics with identified profiles in our sample, descriptive analyses, presented in Table 5, were performed for the educational level variable, which is the highest level of education that a person has successfully completed. The reduced sample size in some profiles did not allow the authors to perform statistical analyses to verify the associations between this variable and the identified profiles.

**Table 5:** Identified interaction quality profiles and participants’ training

Diploma	HQ	MHQ	MQ	MLQ	Total
AEC	6	5	2	1	14
DEC	11	7	1	0	19
University certificate	1	1	3	2	7
Baccalaureate	0	1	3	4	8
Masters	1	3	18	11	33
Total	19	17	27	18	81

Profile affiliation appears to be related to participants’ education level as the highest quality profile (HQ) shows a very high proportion of college education (89.5%), while the lower quality profiles display a majority of graduate degrees, namely, 77.8% for the MQ profile and 83.3% for the MLQ profile. However, caution should be exercised in interpreting these results as education may be strongly linked to socio-political contexts, as each state has specific regulations regarding training requirements. Moreover, inferential analyses between education and profiles could not be conducted due to the small number of participants in some conditions.

Regarding group size and participants’ age, the results of the descriptive analyses are shown in Table 6.

**Table 6:** Group size and participant’s age in identified profiles

Variable	HQ		MHQ		MQ		MLQ	
	M	SD	M	SD	M	SD	M	SD
Group size	9.84	3.72	9.88	4.09	18.67	5.32	20.83	5.7
Age	38.84	8.2	41.18	9.93	46.56	7.67	49.61	4.59

Results show that group size and participants’ age gradually increase from the HQ profile to the MLQ profile, which could suggest that higher quality is linked to lower adult–child ratio and younger participants. To explore this hypothesis, a Kruskal–Wallis test was used to verify whether the profiles were significantly associated with these variables (Table 7).

**Table 7:** Kruskal–Wallis test results

Variable	Kruskal–Wallis’ *H*	DOF	Sig
Group size	41.769	3	< 0.001
Age	17.465	3	0.001

Results shown in Table 7 indicate that tests for both variables were significant and that profiles predict structural characteristics. Analysis of these results using a Mann–Whitney test with Bonferroni’s correction allows the authors to conclude that group size of participants in the HQ and MHQ profiles are significantly lower than that of participants in the MQ and MLQ profiles (*p* ≤001). This suggests that higher classroom quality is associated with lower adult–child ratio. As for participants’ age, the same tests show that participants’ age in the HQ profile is significantly lower than those in the MQ and MLQ profiles (*p* = 0.018 and *p* = 0.001), while participants in the MHQ profile are significantly younger than those in the MLQ profile (*p* = 0.041). In other words, while there is no significant difference between the HQ and MHQ profile, and the MHQ and MQ profiles in terms of age, participants in higher quality profiles generally tend to be younger than those in lower quality profiles.

## Discussion

This study aimed at identifying latent adult–child interaction quality profiles among Quebec ECC educators and French kindergarten teachers and verifying their associations with structural characteristics, namely, educational level, participants’ age, and group size. In line with the authors’ hypothesis, four profiles were identified in this study’s multi-group sample.

Regarding profiles’ scores of adult–child interaction quality, profiles showed scores of emotional support, and classroom organization varying between high and medium quality levels, while instructional quality remained in the low level at the exception of the HQ profile. This suggests that latter domain might need improvement regardless of other variables, such as socio-political context, which is consistent with Pianta et al. (2008) and previous studies. On this subject, socio-political context was found to be significantly associated with interaction quality profiles, with participants from Quebec gathering in the two highest quality profiles and participants form France in the two lowest. This is coherent with Bigras et al.’s (2020) results showing average scores in the Quebec sample generally higher than those of France and suggests that attaining higher educational quality is easier under Quebec’s ECEC policies. In that respect, French stakeholders could benefit from seeking inspiration in Quebec’s ECEC policies as a way to enhance their own quality of services. Some aspects are discussed below regarding structural variables.

In addition, as was expected, most of Quebec participants in the present study gather in the highest quality profile, as is the case with studies in Finland (Salminen et al. 2012) and Vietnam (Hoang et al. 2019), while the majority of French teachers are grouped in a median profile, as for studies carried out in the United States (LoCasale-Crouch et al. 2007) and China (Hu et al. 2016). These results also support Bigras et al.’s (2020) findings in regard to the higher average quality of Quebec’s ECC compared to French kindergartens. They illustrate the need for other studies addressing prevalence heterogeneity in identified profiles according to socio-political context with analogous or dissimilar cultures.

However, it should be pointed out that the CLASS Pre-K (Pianta et al. 2008) is a North American tool with a conceptualization of quality contextualized in a particular culture. As some authors have mentioned, positivist quality assessment tools may prove biased and unrepresentative of quality in other cultural contexts (Samuelsson et al. 2006; Sheridan 2009). In that regard, the CLASS Pre-K could be promoting specific aspects of Quebec’s educational approach, which targets the holistic development of the child, at the expense of the French approach, which emphasizes school readiness (Bigras et al. 2020). This stresses the importance of more international studies analyzing the representativeness of the CLASS scores, and other measurement tools, outside the North American context, and in particular in France, in order to draw adequate conclusions. It also raises the question whether measurement tools assessing aspects of educational quality other than interaction quality, such as pedagogical orientations, would allow us to target strengths in the French approach that could be transposed to other socio-political contexts. Further studies are required to answer this question.

Pertaining to associations between interaction quality profiles and structural quality characteristics, analyses were limited by the small sample size in terms of participants’ training. This constitutes the major limitation of the present study, encompassing the impossibility of carrying out statistical analyzes and lack of power. Descriptive analyses showed that participants in the highest quality profiles (HQ and MHQ) tended to have a collegial degree, while those in the MQ and MLQ profiles had mostly graduate degrees. Yet, because socio-political context turned out to be a predictive variable for quality profile, within the framework of this study, education level might not necessarily be predictive of quality profiles. Indeed, collegial education is sufficient in Quebec to work as an educator in ECC, while university studies are necessary in France to occupy a position in kindergartens. Analyses have shown that the HQ and MHQ profiles gather more participants from Quebec rather than France, but also more participants with a collegial degree as the highest completed training rather than a university degree. However, the association between these two variables might not be bidirectional, as holders of a collegial degree can only work in Quebec’s ECC, France’s education requirement being higher, while Quebec educators might have pursued higher studies while still being able to work in Quebec’s ECC. Hence, because education and training stem from government policies based on current socio-political contexts, the latter could be a stronger indicator of interaction quality as it impacts higher decision processes.

On another note, it seems relevant to mention that French teacher’s master’s degree did not include specific courses on young children’s development and needs (Bigras et al. 2020). Even though Hu et al. (2016) found no association between specialized early childhood training and educational quality, improving French teachers’ initial training with added mandatory child development courses could prove to be an interesting avenue to explore to improve quality in kindergartens. In the same way, educators’ initial training in Quebec requires them to carry out more than 300 h of internship in actual ECS settings. Thus, French teachers in initial training could also carry out part of their internships in kindergarten classrooms in order to assimilate specific knowledge related to young children’s needs and improve the quality of their educational practices with youths.

Finally, analyses found significant associations between profiles, group size, and participants’ age. Indeed, results showed that group size was significantly lower in the HQ and MHQ profiles. Consequently, it can be hypothesized that reducing the group size of French kindergarten classrooms would allow them to offer a higher level of quality. These results are in line with those of LoCasale-Crouch et al. (2007), who reported a generally lower adult–child ratio for higher quality profiles. Again, however, caution should be exercised as regulations regarding adult–child ratio differ between socio-political contexts. Indeed, the maximum number of children per adult in Quebec’s ECC is 8, while it is 22 in France’s kindergartens. Nonetheless, experimental approaches to reducing class sizes could be integrated in France’s ECEC policies to try to raise quality to higher levels.

As for participants’ age, it appears to be the only variable in this study that is not directly linked to socio-political context. Analyses showed that participants in higher quality profiles tend to be younger than those in the lower quality profiles. While this is hard to interpret, one could hypothesize that younger teachers or educators have a more open disposition towards new and up-to-date practices. Therefore, they could have more chances of adopting quality practices (McKenzie et al. 2005). However, this hypothesis needs to be further explored to offer a concrete answer.

## Conclusion

To conclude, this study aimed at identifying latent interaction quality profiles in Quebec’s ECC educators and France’s kindergarten teachers, while investigating their relations to socio-political context and structural variables. Results revealed four profiles of interaction quality in our multi-group sample, showing higher average interaction quality scores for all CLASS domains in Quebec’s ECC compared to kindergartens in France. This points to a more favorable socio-political context for interaction quality in Quebec ECC than in French kindergartens. Results also showed that group size was significantly associated with interaction quality profiles, with higher interaction quality profiles showing lower average adult–child ratio. Hence, group size reduction could be an interesting avenue to explore in France’s ECEC policies for a significant improvement in the average quality in all CLASS domains. Finally, participants’ age was found to be significantly associated with identified profiles, illustrating a general trend of younger participants being significantly more classified in higher quality profiles. It is probable that this might be a sign of younger teachers or educators using more modern practices, increasing higher quality likelihood. Other studies are nonetheless required for one to confirm this hypothesis.

## Data Availability

The datasets analyzed during the current study are available from the corresponding author on reasonable request.
